# Histone lactylation modification promotes docetaxel resistance and tumor progression through CNN1-Mediated autophagy and cell cycle arrest in Castration-resistant prostate cancer

**DOI:** 10.1038/s41420-026-03141-8

**Published:** 2026-05-13

**Authors:** Ruijie Mao, Xu Chen, Xing Fu, Dingyuan Yang, Wenqiang Chen, Jun Li

**Affiliations:** 1 Department of Urology, North Sichuan Medical College, Nanchong, 637000 China; 2 Department of Urology, Sichuan Provincial People’s Hospital, University of Electronic Science and Technology of China, Chengdu, 610072 China; 3 Department of Urology, Chengdu Xinjin District People’s Hospital, Chengdu, 611430 China; 4 Department of Urology, Chengdu No.2 People’s Hospital, Chengdu, 610021 China; 5 Department of Urology, Yibin No.2 People’s Hospital, Yibin, 644000 China

**Keywords:** Prostate cancer, Autophagy, Post-translational modifications

## Abstract

Castration-resistant prostate cancer (CRPC) constitutes an advanced stage of prostate cancer (PCa) that emerges following conventional androgen deprivation therapy (ADT). Docetaxel (DTX), a standard chemotherapeutic agent, is integral to the therapeutic regimen for CRPC. However, the development of resistance to DTX has significantly impeded its clinical efficacy. Histone lactylation and elevated lactate production are emerging as critical factors in cancer biology, yet their roles in CRPC and DTX resistance remain poorly understood. This study investigated the relationship between histone lactylation, lactate production, and DTX resistance in CRPC. Clinical analysis revealed significantly increased pan-lactylated protein (Pan Kla) expression in CRPC tissues compared to PCa, accompanied by elevated lactate production and lactate dehydrogenase (LDH) activity. Higher Pan Kla expression was linked to poor prognosis in CRPC. DTX-resistant CRPC (CRPC-R) samples exhibited significantly elevated Pan Kla and histone lactylation modifications, especially at H3K18la and H4K12la sites. Inhibition of lactate production using 2-deoxyglucose (2-DG) and oxamate reduced DTX resistance, suppressed cell migration, induced G0/G1 phase arrest, and promoted autophagy. Moreover, CNN1 was identified as a potential downstream target of histone lactylation modifications in CRPC. Elevated CNN1 expression correlated with increased lactylation and DTX resistance, whereas its inhibition reversed the effects of lactate inhibition on cell cycle progression and autophagy. In vivo, CNN1 overexpression counteracted the tumor-suppressive effects of lactate inhibition, restoring tumor growth and autophagy levels. These findings suggested that histone lactylation and lactate metabolism, mediated by CNN1, play a crucial role in DTX resistance and tumor progression in CRPC, offering potential therapeutic targets for overcoming chemoresistance in CRPC.

## Introduction

Prostate cancer (PCa) is one of the most common malignancies in men, particularly affecting older individuals [1, 2]. Despite advancements in treatment strategies, castration therapy has remained a standard approach for prostate cancer management, which effectively reduces testosterone levels and controls tumor growth [3]. However, as the disease progresses, PCa cells gradually develop resistance to conventional androgen deprivation therapies, leading to the emergence of castration-resistant prostate cancer (CRPC) [4]. CRPC not only loses sensitivity to androgen deprivation (such as LHRH agonists and anti-androgens), but also undergoes significant biological changes, including increased cell proliferation, enhanced invasiveness, and increased metastatic potential [5]. Docetaxel, one of the standard chemotherapies for CRPC, initially shows efficacy, but resistance develops over time, significantly affecting therapeutic outcomes and patient prognosis [6]. Therefore, further investigation into the molecular mechanisms of CRPC, the identification of new drug targets, and the development of personalized treatment strategies remain critical areas of focus in prostate cancer research.

The mechanisms underlying CRPC drug resistance are complex and multifactorial. These include alterations in the androgen receptor (AR) signaling pathway, remodeling of the tumor microenvironment, and abnormal signaling pathways within cancer cells [7, 8]. In recent years, increasing attention has been focused on the role of epigenetics and transcriptional regulation in CRPC drug resistance [9, 10]. Histone lactylation, a novel form of epigenetic modification, involves the covalent attachment of a lactate molecule to the lysine residue of histones [11]. This modification reflects changes in cellular metabolic states and is closely associated with tumorigenesis and tumor progression [12]. Tumor cells often exhibit a metabolic shift toward aerobic glycolysis, resulting in the accumulation of lactate [13]. Recent reports indicate that enhanced aerobic glycolysis in CRPC promotes the development of chemoresistance in this disease [14]. PCa displays distinct metabolic and bioenergetic profiles based on the disease stage [15]. A prominent example of this metabolic plasticity is the metabolic shift from oxidative phosphorylation to glycolytic metabolism, which frequently accompanies the transition to advanced-stage PCa [15]. Beyond being a metabolic byproduct, lactate also functions as a signaling molecule, modulating the expression of key genes involved in cell proliferation, apoptosis, migration, and immune evasion [16]. The import of lactate into prostate cancer (PCa) cells via monocarboxylate transporter 1 (MCT1) has been demonstrated to stabilize hypoxia-inducible factor 1-alpha (HIF1α) under normoxic conditions through lactylation, thereby acting as a transcriptional enhancer for KIAA1199 [17]. Silencing KIAA1199 selectively impedes angiogenesis and vasculogenic mimicry (VM) in PCa by disrupting hyaluronic acid (HA)-mediated vascular endothelial growth factor A (VEGFA) signaling [17]. CircXRN2 hinders bladder cancer advancement by binding with LAST1, shielding it from SPOP-induced ubiquitylation and degradation and this interaction activates the Hippo signaling pathway, which in turn suppresses H3K18 lactylation [18]. Colorectal cancer (CRC) patients exhibiting resistance to bevacizumab therapy demonstrated increased levels of histone lactylation [19]. Histone lactylation promoted RUBCNL/Pacer transcription, aiding autophagosome maturation by interacting with BECN1 and facilitating the recruitment and activity of the class III phosphatidylinositol 3-kinase complex [19]. The specific functions and mechanisms of histone lactylation in drug resistance and tumor progression in CRPC remain unclear.

Calponin 1 (CNN1), alternatively referred to as basic calcineurin or calcineurin h1, is a 34 kDa troponin-like protein that serves as a marker for the differentiation of cardiac and smooth muscle tissues [20]. It represents one of the three subtypes of calponin and is encoded by a gene located on human chromosome 19 (19p13.2-p13.1) [21]. CNN1 exhibits diverse expression patterns and functions across different types of cancers, suggesting that it may play a complex role in tumor initiation, progression, and metastasis [22]. The expression of CNN1 was higher in normal tissues compared to cancerous tissues. However, some studies indicate that as tumors progress, the expression levels increase again [22]. Elevated expression of calponin 1 (CNN1) in cancer-associated fibroblasts (CAFs) is associated with poor clinical outcomes in gastric cancer (GC) patients, particularly those undergoing 5-Fluorouracil (5-Fu) treatment [23]. CNN1 facilitates CAF-induced extracellular matrix remodeling and stiffening by promoting CAF contraction through a ROCK/MLC-dependent mechanism [23]. Recent studies employing weighted gene co-expression network analysis (WGCNA) in colorectal cancer (CRC) consistently identify CNN1 as a direct facilitator of cancer progression [24]. This facilitation occurs through the induction of epithelial–mesenchymal transition in tumor cells, a process associated with CRC recurrence [24]. The expression of CNN1 in CRPC and its associated regulatory mechanisms remain unclear.

## Results

### Elevated histone lactylation and lactate production were associated with docetaxel resistance and poor prognosis in CRPC

**Fig. 1:**
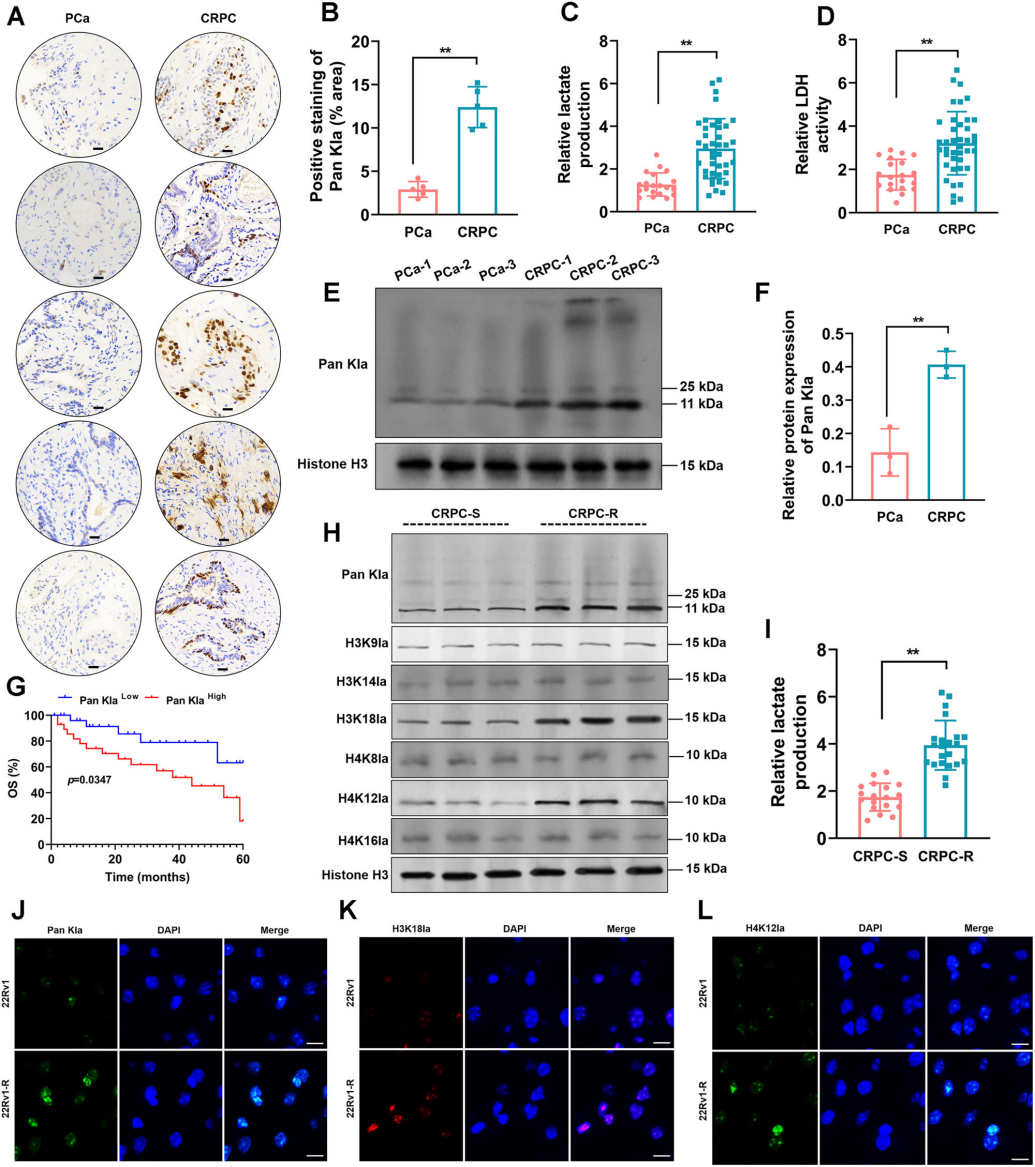
Elevated histone lactylation and lactate production were associated with docetaxel resistance and poor prognosis in CRPC. **A** Immunohistochemical detection of the expression of Pan Kla in clinical PCa and CRPC tissues (*N* = 5). Scale bar=50 μm. **B** Quantitative analysis of Pan Kla-positive staining areas. **C, D** Detection of lactate production levels and LDH activity in clinical PCa (*N* = 20) and CRPC (*N* = 40) samples using the commercial assay kits. **E** Western blot analysis was used to examine the expression of Pan Kla in clinical tissues (*N* = 3). Histone H3 was used as internal reference. **F** Quantitative analysis of expression level of Pan Kla. **G** CRPC samples were classified into high and low Pan Kla expression groups, and survival analysis showed that the high Pan-Kla expression group exhibited poorer prognosis. **H** CRPC tissue samples exhibiting either resistance (CRPC-R) or sensitivity (CRPC-S) to DTX were selected and Western blotting was applied to quantify the levels of Pan Kla and to assess the expression of specific lactylation modification sites (H3K9la, H3K14la, H3K18la, H4K8la, H4K12la and H4K16la) (*N* = 3). **I** Lactate production in CRPC-S (*N* = 18) and CRPC-R (*N* = 22) tissues were measured using a commercial assay kit. **J–L** Immunofluorescence assay detected the co-localization of Pan Kla, H3K18la, and H4K12la with the nuclear marker DAPI in 22Rv1 and 22Rv1-R cells (*N* = 3). Scale bar=20 μm.**p < 0.01. All the data were presented as the means ± SD.

Immunohistochemical staining was performed to assess Pan Kla expression in PCa and CRPC tissues, thereby evaluating lactylation levels in clinical samples. The results (Fig. 1A, B) demonstrated a significant increase in Pan Kla expression in CRPC tissues compared to PCa tissues. Lactate production and LDH activity were assessed using commercial assay kits. Figure 1C, D demonstrated a significant increase in lactate production and LDH activity in CRPC tissues compared to PCa tissues. The expression levels of Pan Kla protein in clinical samples were assessed by Western blot, followed by quantitative analysis. The results of Fig. 1E, F demonstrated a marked increase in Pan Kla expression in CRPC tissues compared to PCa tissues. Subsequently, CRPC samples were stratified into cohorts exhibiting high and low levels of Pan Kla expression for survival analysis. The results (Fig. 1G) demonstrated that elevated Pan Kla expression correlates with poor prognosis. Furthermore, we performed a targeted Western Blot screening panel. As shown in the Fig. 1H and Fig. S2, Western blot was performed to screen of a panel of lactylation sites in DTX-sensitive (CRPC-S) and DTX-resistant (CRPC-R) samples. Our results demonstrated that while the expressions of H3K9la, H3K14la, H4K8la, H4K16la did not changed significantly, H3K18la and H4K12la were the most dramatically upregulated modifications in the resistant phenotype. The results (Fig. 1I) showed that lactate generation were significantly promoted in CRPC-R group compared to the CRPC-S group. Moreover, immunofluorescence assay (Fig. 1J–L) demonstrated that Pan Kla, H3K18la, and H4K12la signals are primarily localized in the nucleus (co-localized with nuclear marker DAPI) in both 22Rv1 and 22Rv1-R cells. These findings suggested that elevated lactate production and histone lactylation modifications in CRPC are associated with DTX resistance and poor prognosis.

### Lactate inhibition attenuated docetaxel resistance, induced G0/G1 arrest, and promoted autophagy in CRPC

**Fig. 2:**
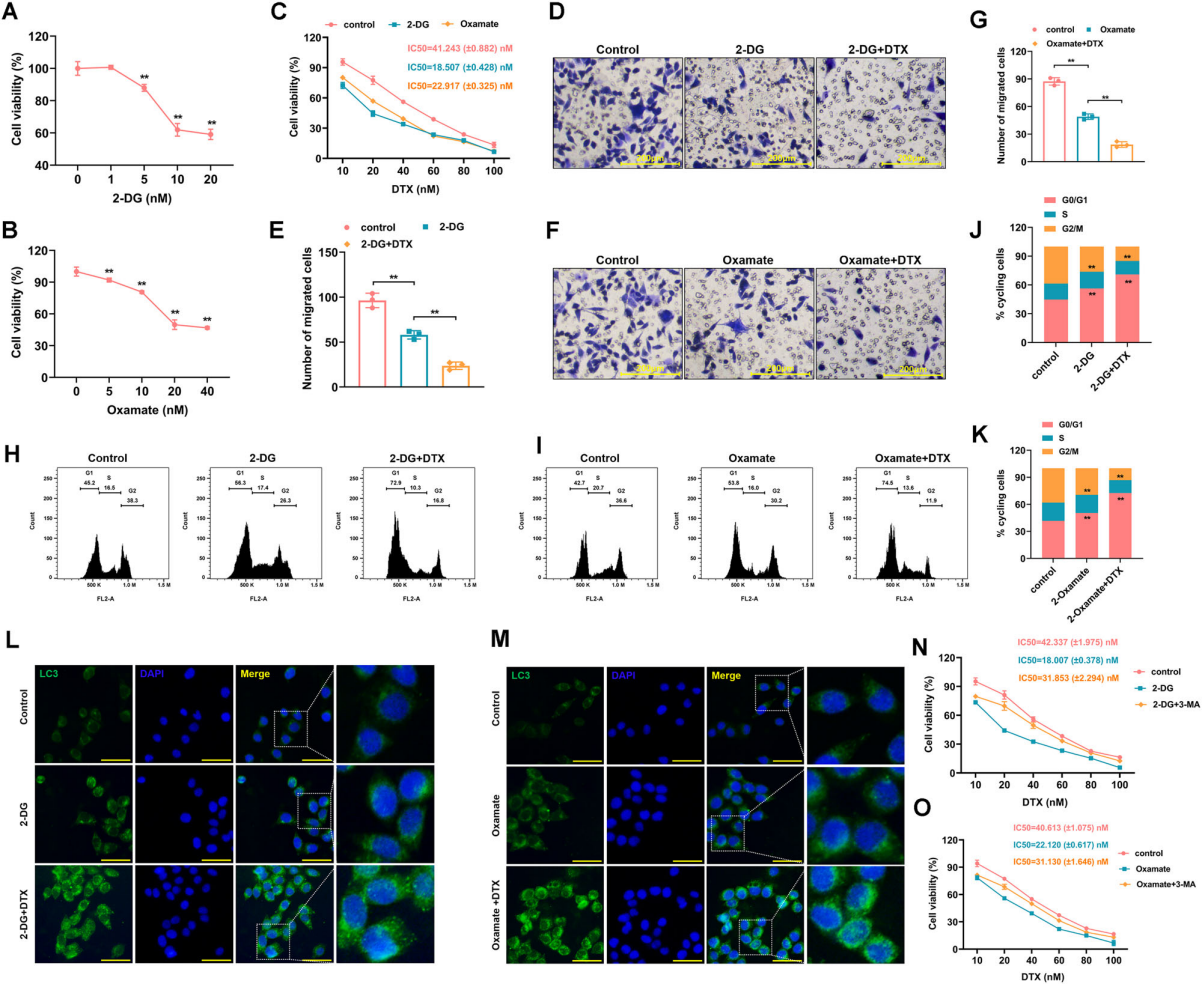
Lactate inhibition attenuated docetaxel resistance, induced G0/G1 arrest, and promoted autophagy in CRPC. **A, B** 22Rv1-R cells were treated with a concentration gradient of 2-DG (0, 1, 5, 10, 20 nM) or oxamate (0, 5, 10, 20, 40 nM), and cell viability was assessed using the CCK-8 assay (*N* = 3). **C** 22Rv1-R cells were treated with 2-DG (10 nM) or oxamate (20 nM) in combination with a concentration gradient of DTX (0, 10, 20, 40, 60, 80, 100 nM) for 48 h and the IC50 value of DTX were determined. **D–G** Transwell assay was conducted to assess the migrated cells (*N* = 3). Scale bar=200 μm. **H–K** cell cycle distribution was determined via a flow cytometer (*N* = 3). **L, M** Immunofluorescence staining was performed to detect LC3 fluorescence expression in cells to assess the level of autophagy (*N* = 3). Cell nuclei were stained with DAPI. Scale bar=50 μm. **N, O** 22Rv1-R cells were treated with 3-MA combined with 2-DG or oxamate, and cell viability was assessed using the CCK-8 assay (*N* = 3). **p < 0.01. All the data were presented as the means ± SD.

To investigate the impact of lactylation modifications on the biological functions of CRPC cells, the lactate production inhibitors of 2-DG and oxamate were applied to treat 22Rv1-R cells. CCK-8 assays were performed and the results (Fig. 2A, B) showed that cell viability was gradually inhibited as the concentrations of 2-DG and oxamate increased. When 2-DG and oxamate were combined with a concentration gradient of DTX, the addition of 2-DG or oxamate remarkably reduced the IC50 value of DTX and decreasing DTX resistance (Fig. 2C). Additionally, transwell assay was conducted to evaluate cell migration, revealing that treatment with 2-DG or oxamate significantly attenuated cell migration (Fig. 2D–G). Moreover, the combination of 2-DG or oxamate with DTX resulted in a more pronounced inhibition of cell migration. Treatment with 2-DG or oxamate obviously promoted the proportion of cells in the G0/G1 phase with attenuating the proportion in G2/M phase, while the combination with DTX and 2-DG or oxamate further enhanced G0/G1 phase arrest (Fig. 2H–K). Moreover, immunofluorescence staining was carried out to evaluate LC3 expression as a measure of autophagy levels. The administration of 2-DG or oxamate resulted in a significant upregulation of LC3 expression, suggesting inhibition of lactate facilitated the induction of autophagy (Fig. 2L, M, Fig. S3). Moreover, when used in combination with DTX, 2-DG or oxamate further augmented LC3 expression, thereby enhancing autophagy level. To validate the critical role of autophagy, we performed rescue experiments using 3-MA (an autophagy inhibitor). The results of Fig. 2N, O showed that co-treatment with 3-MA reversed the effects of 2-DG/oxamate with increasing DTX IC50 value (restored DTX resistance). This data confirmed that autophagy is a critical mediator of lactate inhibition-induced DTX sensitivity.

### Identification of CNN1 as a downstream target of histone lactylation modification in CRPC

**Fig. 3:**
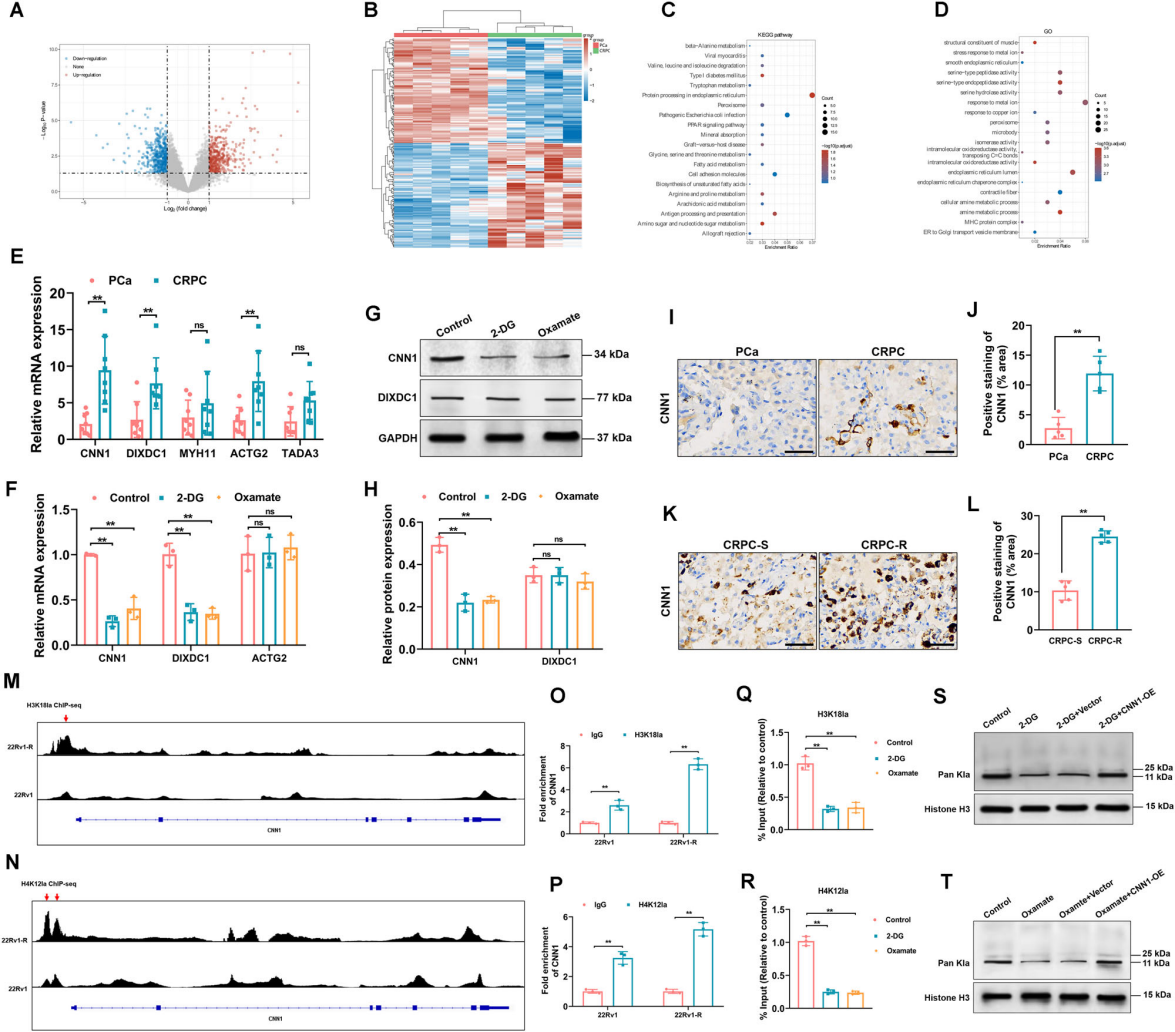
Identification of CNN1 as a downstream target of histone lactylation modification in 22Rv1-R cells. The volcano map **A** and heatmap (**B**) of differentially expressed mRNAs in PCa and CRPC samples from GEO dataset (GSE32269). **C, D** Kyoto Encyclopedia of Genes and Genomes (KEGG) and Gene Ontology (GO) pathway analyses of differentially expressed genes. **E** RT-PCR validation of the top 5 upregulated genes (CNN1, DIXDC1, MYH11, ACTG2, TADA3) in PCa and CRPC samples (*N* = 3). **F** 22Rv1-R cells were treated with 2-DG and Oxamate, and RT-PCR was used to assess the expression of CNN1, DIXDC1, and ACTG2 (*N* = 3). **G**, **H** Western blot analysis and quantitative analysis of expression of CNN1 and DIXDC1 (*N* = 3). **I, J** Immunohistochemical analysis of CNN1 expression in PCa and CRPC tissues. Scale bar=50 μm. **K**, **L** Immunohistochemical analysis of CNN1 expression in CRPC-S and CRPC-R tissues. Scale bar=50 μm. **M, N** IGV (Integrative Genomics Viewer) peak tracks showing H3K18la and H4K12la peaks at the CNN1 promoter region in 22Rv1 and 22Rv1-R cells. **O, P** ChIP-qPCR validation to quantify the enrichment of H3K18la and H4K12la at the CNN1 promoter (*N* = 3). **Q**, **R** ChIP-qPCR was performed to assess the enrichment of H3K18la and H4K12la at the CNN1 gene promoter (*N* = 3). **S**, **T** 22Rv1-R cells were infected with the CNN1-OE lentivirus, and Western blot analysis was used to detect the Pan Kla protein expression levels (*N* = 3). ns=non-significant, **p < 0.01. All the data were presented as the means ± SD.

To further investigate the molecular mechanisms underlying lactylation modification on DTX resistance in CRPC, we performed GEO data analysis to identify differentially expressed genes between PCa and CRPC samples (Fig.3A, B). Subsequently, KEGG and GO enrichment analyses were conducted on the upregulated genes in CRPC samples to gain insights into the potential pathways and biological processes involved (Fig.3C, D). The top-five upregulated genes in CRPC (CNN1, DIXDC1, MYH11, ACTG2, TADA3) were validated through RT-PCR analysis. As represented in Fig.3E, compared to the PCa group, the expression of CNN1, DIXDC1, and ACTG2 was significantly upregulated in CRPC samples. Furthermore, treatment with 2-DG or oxamate remarkably reduced the mRNA levels of CNN1 and DIXDC1 (Fig.3F) and suppressed the protein expression of CNN1 in 22Rv1-R cells (Fig.3G, H), thus selecting CNN1 as the subject for further investigation. In addition, Immunohistochemistry was performed to detect the expression of CNN1 in clinical PCa and CRPC samples, as well as in CRPC DTX-resistant and DTX-sensitive samples. The results demonstrated that, the expression of CNN1 was significantly elevated in the CRPC group compared to PCa samples (Fig.3I, J), as well as in the CRPC DTX-resistant samples compared to DTX-sensitive samples (Fig.3K–L). Moreover, IGV (Integrative Genomics Viewer) peak tracks represented H3K18la and H4K12la peaks at the CNN1 promoter region in 22Rv1 and 22Rv1-R cells (Fig.3M, N). The IGV tracks clearly showed that H3K18la and H4K12la peaks are significantly higher in 22Rv1-R cells than in 22Rv1 cells, and these peaks are located within the CNN1 promoter region. Besides, we performed ChIP-qPCR validation to quantify the enrichment of H3K18la and H4K12la at the CNN1 promoter. The results of Fig.3O, P showed that H3K18la and H4K12la were enriched in 22Rv1 and 22Rv1-R cells, and the enrichment of 22Rv1-R cells were higher than 22Rv1 cells. Furthermore, ChIP-qPCR identified the enrichment of H3K18la and H4K12la at the CNN1 gene promoter, which was notably reduced by the treatment of 2-DG and oxamate (Fig.3Q, R). CNN1 overexpression (CNN1-OE) significantly reversed the inhibitive impacts of 2-DG or Oxamate on histone lactylation levels in 22Rv1-R cells (Fig.3S, T, Fig. S4).

### Histone lactylation modulated DTX resistance, autophagy and cell cycle arrest in CRPC through CNN1

**Fig. 4:**
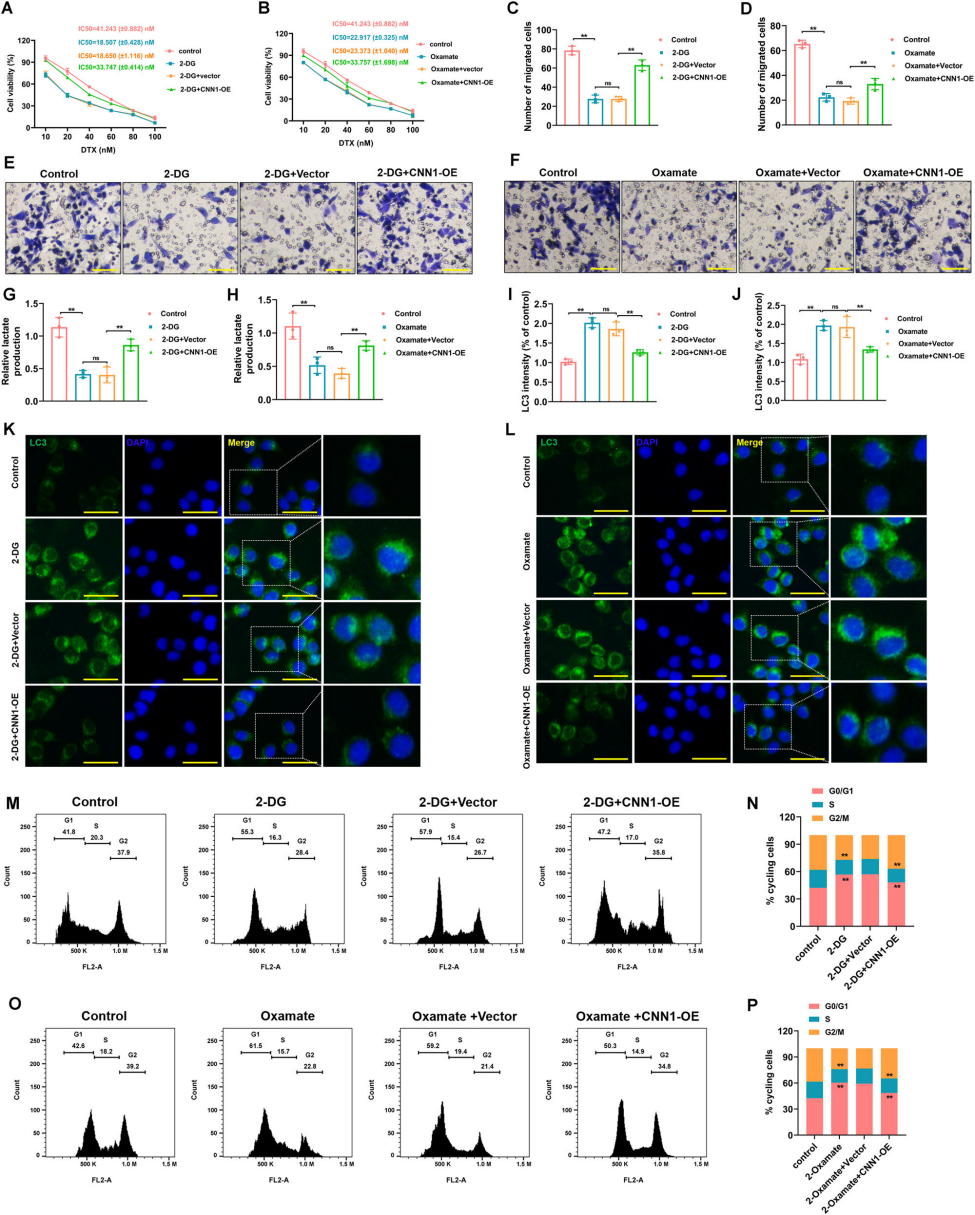
Histone lactylation modulated DTX resistance, autophagy and cell cycle arrest in CRPC through CNN1. **A, B** The cell viability of 22Rv1-R cells and the IC50 value of DTX were measured using CCK-8 assay (*N* = 3). **C–F** Transwell assay was conducted to assess the migrated cells (*N* = 3). Scale bar=200 μm. **G**, **H** Detection of lactate production levels in cells using the commercial assay kit (*N* = 3). **I–L** Immunofluorescence staining was applied to detect LC3 fluorescence expression in cells (*N* = 3). Cell nuclei were stained with DAPI. Scale bar=50 μm. **M–P** Cell cycle distribution was determined via a flow cytometer (*N* = 3). ns=non-significant, **p < 0.01. All the data were presented as the means ± SD.

To examine the roles of CNN1 in regulating DTX resistance, the rescue experiments were performed and CCK-8 assay results showed that overexpression of CNN1 effectively reversed the effects of 2-DG and oxamate, promoting cell viability and increasing the DTX IC50 value, which in turn restored DTX resistance in 22Rv1-R cells (Fig.4A, B). Furthermore, our data showed that CNN1 overexpression effectively reversed the inhibitory effects of 2-DG or oxamate on cell migration (Fig.4C, F) and lactate production (Fig.4G, H). Additionally, treatment with 2-DG or oxamate significantly increased the expression of LC3 in 22Rv1-R cells. In contrast, CNN1 overexpression effectively reversed these effects, resulting in a marked decrease in LC3 expression and inhibition of autophagy (Fig.4I–L). Moreover, CNN1 overexpression remarkably reversed the impacts of 2-DG or oxamate, alleviating the cell cycle arrest with decreasing in the G0/G1 phase (Fig.4M–P). These findings suggested that CNN1 mediated the lactylation modification-regulated autophagy and cell cycle arrest in CRPC.

### CNN1 overexpression reversed the histone lactylation mediated regulation of autophagy and tumor progression in CRPC

**Fig. 5:**
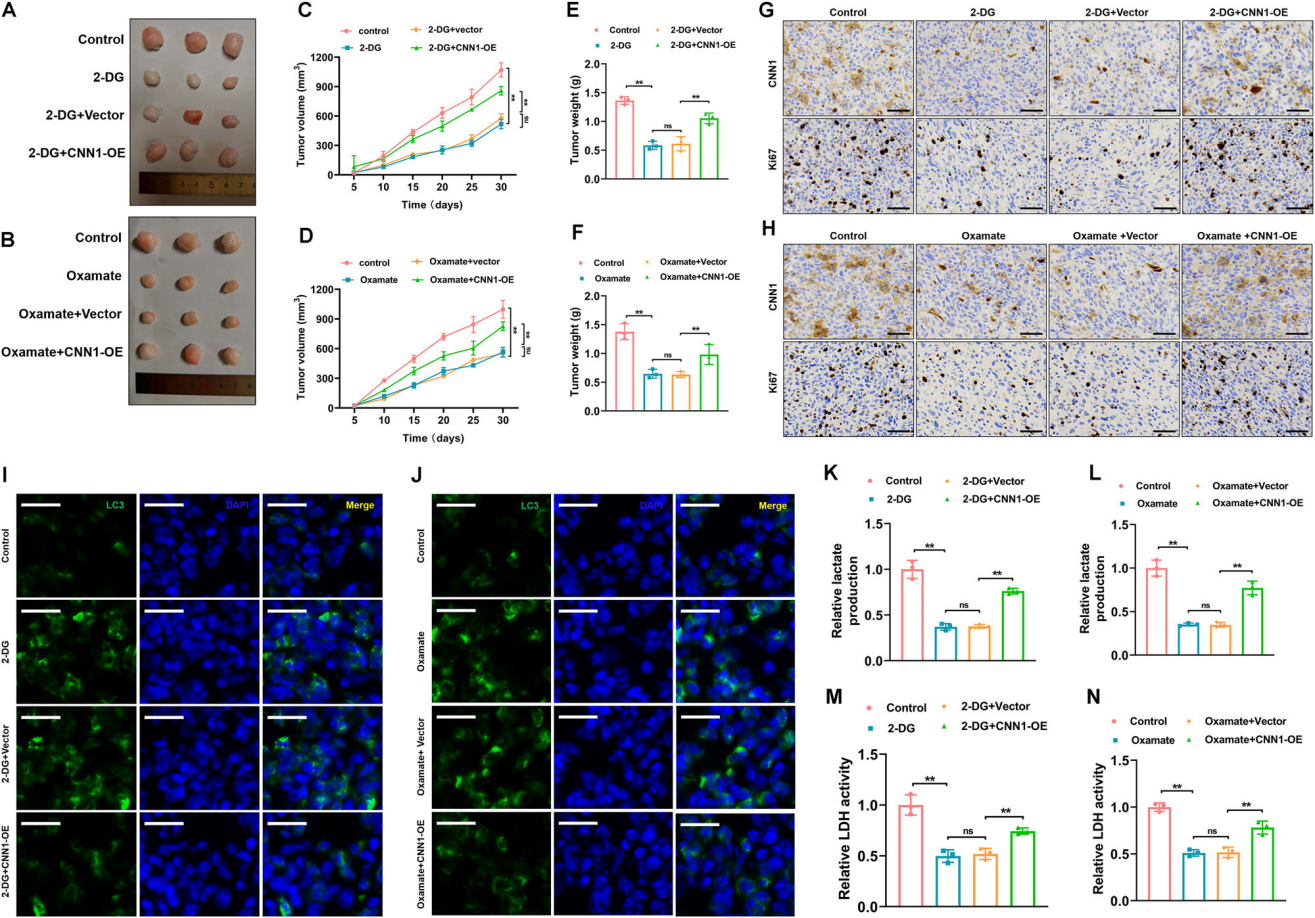
CNN1 overexpression reversed the histone lactylation mediated regulation of autophagy and tumor progression in CRPC. **A, B** Morphology of 22Rv1-R transplanted tumor in nude mice *N* = 3). Tumor weights **C, D** and volumes **E, F** were measured. **G**, **H** Immunohistochemical analysis of CNN1 and Ki67 expression in xenograft tumor samples (*N* = 3). Scale bar=50 μm. **I, J** Immunofluorescence staining was carried out to detect LC3 fluorescence expression in tumor tissues (*N* = 3). Cell nuclei were stained with DAPI. Scale bar=50 μm. **K, L** Lactate levels in tumor tissues were measured using the assay kit (*N* = 3). **M, N** LDH activity in tumor tissue samples was measured via the assay kit (*N* = 3). ns non-significant, **p < 0.01. All the data were presented as the means ± SD.

In vivo experiments were subsequently performed to confirm the in vitro assay findings. Tumor progression was assessed on a weekly basis, and upon conclusion of the experimental period, tumors were excised, weighed, and documented through photography. The results demonstrated that administering 2-DG or oxamate significantly inhibited tumor growth compared to the control group (Fig.5A–F). Moreover, the overexpression of CNN1 markedly counteracted the inhibitory effects of 2-DG or oxamate, thereby enhancing the growth of subcutaneous tumors. Immunohistochemical staining was performed to assess CNN1 and Ki67 expression in tumor tissues. The results (Fig.5G, H and Fig. S5) unveiled that treatment with 2-DG or oxamate significantly decreased Ki67 and CNN1 expression in tumor tissues. The overexpression of CNN1 significantly mitigated the impact of 2-DG or Oxamate, leading to a marked increase in the expression levels of Ki67 and CNN1. Immunofluorescence staining was conducted to assess the expression of LC3 in tumor tissues. In alignment with the in vitro results, the overexpression of CNN1 markedly counteracted the effects of 2-DG or oxamate, leading to a decreased expression of LC3 and a subsequent inhibition of autophagy in the tumor tissues (Fig.5I, J). Moreover, the 2-DG/oxamate-suppressed lactate generation and LDH activity were notably reversed by CNN1-OE (Fig.5K–N). The in vivo results aligned with the in vitro findings, indicating that CNN1 plays a role in histone lactylation, which regulates autophagy and tumor progression in CRPC.

## Discussion

This study investigated the roles of histone lactylation in CRPC, particularly in relation to DTX resistance and tumor prognosis. Our experimental findings indicated that elevated histone lactylation was closely associated with DTX resistance and poor prognosis in CRPC, revealing a potential mechanism linking lactate metabolism to cancer drug resistance and providing a novel therapeutic strategy to overcome CRPC resistance.

Lactate, a key intermediate in cellular metabolism, has been recognized as an adaptive mechanism for tumor cells under conditions of hypoxia and high metabolic demand [25]. Lactate modulates the transcriptional activity of several pivotal oncogenes and other driver genes implicated in metabolic reprogramming, cell cycle regulation, and cellular proliferation [26]. Lactate generated by cancer cells increased the proportion of M2-type tumor-associated macrophages (TAMs) and elevated the intracellular levels of high mobility group box 1 (HMGB1) within TAMs, which further activated the ERK, Wnt, and epithelial-mesenchymal transition (EMT) signaling pathways and exacerbating the malignant characteristics of colorectal cancer (CRC) cells [27]. Lactate has been identified as a facilitator of immune evasion in breast cancer cells through the activation of the GPR81 receptor within the tumor microenvironment (TME) [28]. This activation leads to the upregulation of PD-L1 expression and concurrently suppresses the presentation of tumor-specific antigens by antigen-presenting cells to other immune cells [28]. In our study, we observed a significant increase in lactate levels associated with DTX resistance in CRPC cells, suggesting the importance of lactate metabolism in CRPC. Furthermore, our findings demonstrated that lactate inhibitors notably reduced DTX resistance in CRPC cells, induced G0/G1 cell cycle arrest, and promoted autophagy. Recent reports have shown that autophagy plays a critical role in DTX resistance in CRPC [29]. Zeng et al. found that Prostate Leucine Zipper (PrLZ) enhances DTX tolerance in PCa through abrogation of LKB1/AMPK axis-induced autophagic activity [29]. In our study, we observed that lactate inhibition (2-DG/oxamate) promoted autophagy and reduced DTX resistance. Besides, our study observed a mechanistic phenomenon regarding cell cycle and cell death pathways. DTX canonically acts as a microtubule stabilizer, inducing cell death via G2/M arrest [30]. However, DTX-resistant CRPC cells often bypass this checkpoint. We found that lactate inhibition (2-DG or oxamate) induced profound G0/G1 phase arrest. Rather than antagonizing the effects of DTX, we propose that this metabolic blockade triggers severe energy stress, overriding the survival signals of resistant cells and rerouting them from proliferation to cytotoxic autophagy. Indeed, excessive or prolonged autophagy can serve as a non-apoptotic programmed cell death mechanism (autophagic cell death). Our validation with the autophagy inhibitor 3-MA confirmed that dampening autophagy rescued cell viability, demonstrating that lactate inhibition eliminated resistant CRPC cells with DTX through an autophagy-dependent death mechanism.

Histone lactylation, as a novel post-translational modification, has recently gained attention in cancer research [31, 32]. Histone lactylation plays a role in tumorigenesis by enhancing the expression of YTHDF2 and YTHDF2 identifies m6A-modified PER1 and TP53 mRNAs and facilitates their degradation, thereby accelerating the tumorigenesis of ocular melanoma [33]. Lactate production in endometrial carcinoma induced histone lactylation, which subsequently modulated the expression of ubiquitin-specific peptidase 39 (USP39) [34]. USP39, in turn, activated the PI3K/AKT/HIF-1α signaling pathway through its interaction with PGK1, which ultimately enhanced glycolytic activity and further augmented of histone lactylation [34]. In our study, we identified CNN1 as a downstream target of histone lactylation, playing a key role in CRPC drug resistance. A notable elevation in the highest CCN1 score was observed in recurrent tissues of triple-negative/basal-like breast cancer (BC) tumors [35]. The stable silencing of CCN1 in triple-negative/basal-like BC cells resulted in a substantial decrease in the expression of the CCN1 integrin receptor αvβ3, inhibited anchorage-dependent cell proliferation, diminished clonogenic potential, and impaired migratory capacity [35]. Our study revealed that overexpression of CNN1 reversed the effects of histone lactylation on autophagy and tumor progression, indicating that CNN1 interacted with lactylation pathways to regulate DTX resistance.

CRPC remains a significant challenge in PCa treatment, particularly due to the development of resistance to chemotherapy drugs such as DTX [36]. Modulating lactylation and its downstream targets presents a promising approach to overcoming CRPC resistance. Our findings indicated that lactylation and CNN1 may serve as potential therapeutic targets, and the combination with lactate inhibitors or CNN1 modulators could enhance the efficacy of DTX treatment and improve patient prognosis. Further exploration of the mechanisms by which lactylation influences CRPC subtypes will be critical, particularly in how lactylation and metabolic reprogramming contribute to drug resistance. Moreover, given the metabolic background of PCa, we will further explore the regulatory mechanisms underlying CRPC resistance involving lipid-derived modifications, such as palmitoylation. Besides, additional animal model studies are required to validate the biological function and therapeutic potential of lactylation and CNN1 in CRPC. With the increasing focus on immunotherapy and metabolic reprogramming, targeting lactylation and its regulatory network may provide novel insights for improving the CRPC clinical treatments.

## Materials and methods

### Clinical samples and cell culture

The clinical samples including 20 cases of PCa tissues and 40 cases of CRPC tissues were obtained from Sichuan Provincial People’s Hospital. All experiments were approved by the Ethics Committee of Sichuan Provincial People’s Hospital (Approval No.2023-580). Human CRPC (22Rv1) cells were obtained from the Cell Bank of Chinese Academy of Science (Shanghai, China). Cells were kept in RPMI1640 medium (11875093, Gibco, MA, USA) supplemented with 10% fetal bovine serum (FBS, 10099158, Gibco, MA, USA) in a humidified incubator at 37°C and 5% CO_2_ atmosphere. Docetaxel-resistant 22Rv1(22Rv1-R) cells were developed by chronically exposing 22Rv1 cells to progressively increased concentrations of docetaxel (DTX, HY-B0011, MedChemExpress, Shanghai, China). 22Rv1-R cells were maintained with 25 nM DTX. The Oxamate (HY-W013032A, MedChemExpress, Shanghai, China) and 2-DG (HY-13966, MedChemExpress, Shanghai, China) were used to treat 22Rv1-R cells at the indicated concentrations for 48 h, respectively.

### Construction and infection of lentiviral vectors

The CNN1 coding sequence (CDS) was inserted into the LV5-GFP-Puro lentiviral vector (GenePharma, Shanghai, China) to create LV5-GFP-Puro-CNN1 lentiviral vectors (CNN1-OE). Lentivirus was collected 48 h post co-transfection of LV5-GFP-Puro-CNN1 or empty lentiviral vectors with packaging vectors into 293 T cells using Lipofectamine 3000 (L3000001, Invitrogen, Carlsbad, CA, USA). The supernatant was harvested following a 48-h incubation period. The virus titer was measured in the supernatant following filtration and centrifugation, which contained the virus particles. The lentivirus was used to infect 22Rv1-R cells. Overexpression efficiency of CNN1 was determined via RT-PCR analysis (Fig. S1)

### CCK-8 assay

A CCK-8 assay kit (C0038, Beyotime, Shanghai, China) was applied to examine the cell proliferation. 22Rv1-R cells were grown in 96-well plates at a density of 5000 cells/well. After reaching 80% confluence, 22Rv1-R cells were treated with a concentration gradient of 2-DG (0, 1, 5, 10, 20 nM) or oxamate (0, 5, 10, 20, 40 nM) for 48 h. Next, 10 µL CCK-8 solution was introduced into each well and cultured for 2 h at 37°C. Then, the absorbance was detected at 450 nm via a spectrophotometer (Multiskan MK3, Thermo Scientific, Waltham, MA, USA). To assess the resistance of 22Rv1-R cells, the cells were treated with 2-DG (10 nM) or oxamate (20 nM) in combination with a concentration gradient of DTX (0, 10, 20, 40, 60, 80, 100 nM) for 48 h. Cell viability and the IC50 value of DTX were determined following the treatment.

### Measurement of lactate production and LDH activity

Lactate levels in both cells and tissues were quantified using a commercially lactate assay kit (D799099, Sangon, Shanghai, China). Cells were collected and lysed in ice-cold lysis buffer, and protein concentrations in the lysates were determined using a BCA protein assay kit (P0010, Beyotime, Shanghai, China). The tumor tissues were collected and homogenized in ice-cold PBS using a tissue homogenizer. The homogenate was then centrifuged at 12,000 rpm for 10 min at 4°C to remove debris. Lactate concentrations were measured according to the manufacturer’s instructions. LDH activity was detected through a commercial kit (BC0685, Solarbio, Beijing, China) following the instructions of producer. The absorbance was measured using a microplate reader (Multiskan MK3, Thermo Scientific, Waltham, MA, USA) at 450 nm.

### Western blot

Total protein is extracted from cells or tissues, and protein concentration is determined using the BCA kit (P0010, Beyotime, Shanghai, China). Equal amounts (25 μg) of protein samples are mixed with loading buffer and separated by SDS-PAGE electrophoresis. After separation, proteins are transferred to a PVDF membrane (ISEQ00010, Millipore, Billerica, MA, USA) and blocked with 5% non-fat milk for 1 h. The membrane is incubated overnight at 4°C with primary antibodies, including anti-Pan Kla (1:1000 dilution, PTM-1401RM, PTM Bio, Hangzhou, China), Histone H3 (1:3000 dilution, PTM-1001RM, PTM Bio, Hangzhou, China), H3K9la (1:2000 dilution, PTM-1419RM, PTM Bio, Hangzhou, China), H3K14la (1:2000 dilution, PTM-1414RM, PTM Bio, Hangzhou, China), H3K18la (1:2000 dilution, PTM-1406RM, PTM Bio, Hangzhou, China), H4K8la (1:2000 dilution, PTM-1415RM, PTM Bio, Hangzhou, China), H4K12la (1:2000 dilution, PTM-1411RM, PTM Bio, Hangzhou, China), H4K16la (1:2000 dilution, PTM-1417RM, PTM Bio, Hangzhou, China), CNN1 (1:5000 dilution, ab46794, Abcam, Shanghai, China), DIXDC1 (1:2000 dilution, 13816-1-AP, Proteintech, Wuhan, China) and GAPDH (1:1000 dilution, ab263962, Abcam, Shanghai, China). Subsequently, the blots were incubated with HRP conjugated secondary antibodies for 4 h. The signals were observed utilizing ECL reagents (180-5001, Tanon, Shanghai, China) and captured by the Chemiluminescent Imaging System (5200, Tanon, Shanghai, China).

### Transwell assay

The transwell migration assay was conducted using 24-well transwell chambers (Corning Inc.). After the indicated treatment, 22Rv1-R cells (1×10⁵ cells/well) in 100 μL serum-free medium were seeded in the upper chamber, while 500 μL of medium containing 10% FBS was placed in the lower chamber to serve as a chemoattractant. After 12 h, cells that migrated to the underside of the filter were fixed with 4% paraformaldehyde (P0099, Beyotime, Shanghai, China) for 15 min and stained with 0.5% crystal violet (G1062, Solarbio, Beijing, China) for 20 min. Migrated cells were observed using a fluorescence microscope (IX51, Olympus Corporation, Tokyo, Japan).

### Detection of cell cycle

22Rv1-R cells were collected and fixed in 70% cold ethanol, incubated overnight at 4°C. Afterwards, cells are washed twice with PBS to remove residual ethanol. Next, RNA is digested by incubating cells with 500 μL RNase A (20 μg/mL, EN0531, Thermo Scientific, Waltham, MA, USA) at room temperature for 30 min. The cells are then stained with propidium iodide (PI, 50 μg/mL, HY-D0815, MedChemExpress, Shanghai, China) for 30 min to label DNA. Finally, the cell cycle distribution is analyzed using a flow cytometer (FACSVerse, BD Biosciences, San Jose, CA, USA) to determine the proportion of cells in each phase.

### Immunofluorescence staining

Immunofluorescence staining of tissue sections and cells was conducted as previously described. Briefly, the samples were fixed with 4% formaldehyde and then washed three times with PBS. Non-specific binding was blocked using 3% BSA. The sections were incubated overnight with primary LC3 antibody (1:200 dilution, 4108S, Cell Signaling Technology, Shanghai, China), Pan Kla (1:50 dilution, PTM-1401RM, PTM Bio, Hangzhou, China), H3K18la (1:50 dilution, PTM-1406RM, PTM Bio, Hangzhou, China) or H4K12la (1:50 dilution, PTM-1411RM, PTM Bio, Hangzhou, China) followed by incubation with FITC-conjugated secondary antibody (1:500 dilution, Goat Anti-Rabbit IgG (H + L), A0562, Beyotime, Shanghai, China) for 1 h. Fluorescence images were captured using an Olympus IX51 microscope (Olympus Corporation, Tokyo, Japan).

### Bioinformatics analysis

The mRNA expression dataset (GSE32269), comprising five PCa and five CRPC clinical samples, was sourced from the Gene Expression Omnibus (GEO) database (https://www.ncbi.nlm.nih.gov/geo/). Differential analysis was performed using the “edgeR” package in R, identifying differentially expressed genes (DEGs) with a threshold of |log fold change | > 1.5 and *p* < 0.05. The Cluster Profiler package was utilized for KEGG and GO enrichment analysis to investigate the biological processes, cellular components, molecular functions, and signaling pathways associated with upregulated genes in CRPC.

### Reverse transcription polymerase chain reaction (RT-PCR) analysis

Total RNA from tissues were extracted using Trizol (R0016, Beyotime, Shanghai, China) following the manufacturer’s instructions. RNA was converted to cDNA utilizing the SweScript All-in-One RT SuperMix (G3337, Servicebio, Wuhan, China). Real-time RT-PCR was conducted on a BioRad CFX Connect Real-Time PCR System using SYBR Green qPCR Master Mix (G3320, Servicebio, Wuhan, China). The target gene expression level was quantified using the 2^−ΔΔCT^ method. GAPDH was used as an internal control. All primers used in this experiment were listed in Table S1.

### Chromatin immunoprecipitation (ChIP)-qPCR analysis

ChIP-qPCR was conducted following standard procedures. In brief, cells were crosslinked in 1% formaldehyde at room temperature. Immunoprecipitation of DNA was performed from sonicated cell lysates using antibodies against H3K18la (1:500, PTM-1406RM, PTM Bio, Hangzhou, China), H4K12la (1:500, PTM-1411RM, PTM Bio, Hangzhou, China), or IgG, incubating overnight at 4°C. The antibody-chromatin complex was captured by incubating with protein A/G beads (80106 G, Invitrogen, Carlsbad, CA, USA) for 1 h. After washing, the beads were resuspended in ChIP elution buffer, treated with proteinase K (ST535-2g, Beyotime, Shanghai, China) at 65°C for 1.5 h, followed by incubation at 95°C for 10 min to reverse the protein-DNA crosslinks. Ten percent of the sonicated chromatin was reserved as an input control for qPCR analysis. ChIP signals were quantified by quantitative PCR.

### Xenograft tumorigenesis

BALB/c nude mice, aged 6–8 weeks, were acquired from Cavens Experimental Animal Center located in Changzhou, China. The mice were housed in a room with unrestricted access to standard food and water for one week, and randomly divided into two large groups. Group 1: (1) control (*n* = 3), (2) 2-DG (*n* = 3), (3) 2-DG + vector (*n* = 3), and (4) 2-DG + CNN1-OE (*n* = 3). Group 2: (1) control (*n* = 3), (2) oxamate (*n* = 3), (3) oxamate + vector (*n* = 3), and (4) oxamate + CNN1-OE (*n* = 3). In the control, 2-DG and oxamate group, a total of 2 × 10^6^ 22Rv1-R cells were separately injected into the left flank of nude mice. In the other groups, 22Rv1-R cells infected with the vector or CNN1-OE lentivirus were subcutaneously implanted into the nude mice. 2-DG (10 mg/kg per injection) was administered intraperitoneally once daily for 15 consecutive days. Oxamate (300 mg/kg per injection) was administered intraperitoneally twice a week for 15 days. The control group received an equal volume of saline via intraperitoneal injection for the same duration. Tumor size was assessed every 5 days, with volume calculated as (length × width² / 2). After 30 days of subcutaneous cell implantation, all mice were euthanized. Tumor tissues were subsequently collected, rinsed with PBS, and weighed. The Ethics Committee of Sichuan Provincial People’s Hospital (Approval No.2023-580) ensured compliance with all animal handling and experimental procedures, following the guidelines for the Care and Use of Laboratory Animals.

### CNN1 and Ki67 immunohistochemistry staining

Tumor tissues were promptly excised and fixed in 10% buffered formalin. Immunohistochemistry was performed to assess CNN1 and Ki67 expression in the tumor samples. Tissue sections (4 μm) were incubated overnight at 4°C with primary antibodies against CNN1 (1:1000, ab46794, Abcam, Shanghai, China) or Ki67 (1:500, ab15580, Abcam, Shanghai, China). After washing, the sections were incubated with a secondary antibody, horseradish peroxidase (HRP)-conjugated goat anti-rabbit IgG (1:50, A0208, Beyotime, Shanghai, China) for 1 h at room temperature. The sections were then counterstained with hematoxylin to visualize the nuclei and examined using a fluorescence microscope (IX51, Olympus, Tokyo, Japan). Positive staining areas were quantified using ImageJ software.

### Statistical analysis

Data were presented as mean ± standard deviation (SD). Statistical analysis was performed using GraphPad Prism 7.0 (GraphPad Software, CA, USA). Normality and homogeneity of variance were tested. A Student’s t-test was applied for comparisons between two groups, while one-way analysis of variance (ANOVA) followed by Tukey’s post-hoc test was used for comparisons among multiple groups. P-values less than 0.05 were considered statistically significant.

## Supplementary information

Supplemental Material

original images of Western blots

## Data Availability

The data utilized and/or analyzed throughout this research are available from the corresponding author on reasonable request.
