# Supplementary material for: Histone lactylation modification promotes docetaxel resistance and tumor progression through CNN1-Mediated autophagy and cell cycle arrest in Castration-resistant prostate cancer
Source: Cell Death Discov. 2026 May 13;12:259. doi: 10.1038/s41420-026-03141-8 (PMC13221474; doi:10.1038/s41420-026-03141-8)
Supplement: Supplementary file 1 — Supplemental Material [file 41420_2026_3141_MOESM1_ESM.docx]

**Table S1** RT-PCR primer sequences in this study

| CNN1 | Forward | 5ʹ-TTGAGGCCAACGACCTGTTT-3ʹ |
| --- | --- | --- |
|  | Reverse | 5ʹ-CTGGGTACTCGGGAGTCAGA-3ʹ |
| DIXDC1 | Forward | 5ʹ-AGATGAGAGGAACCGGCTCT-3ʹ |
|  | Reverse | 5ʹ-GTACAGGTGCTGCTGACAGT-3ʹ |
| MYH11 | Forward | 5ʹ-CAGCCTGGGCAACGTAGTAA-3ʹ |
|  | Reverse | 5ʹ-CACTTGGTGAACTGTGCGTG-3ʹ |
| ACTG2 | Forward | 5ʹ-TGTGCTCTCCCTCTATGCCT-3ʹ |
|  | Reverse | 5ʹ-AATGCCAGGGTACATGGTGG-3ʹ |
| TADA3 | Forward | 5ʹ-CTGGCAAAGGAGGAGGTGAG-3ʹ |
|  | Reverse | 5ʹ-GGAGACAGGGGTAGGGGATT-3ʹ |
| GAPDH | Forward | 5ʹ-CGACCACTTTGTCAAGCTCA-3ʹ |
|  | Reverse | 5ʹ-AGGGGTCTACATGGCAACTG-3ʹ |


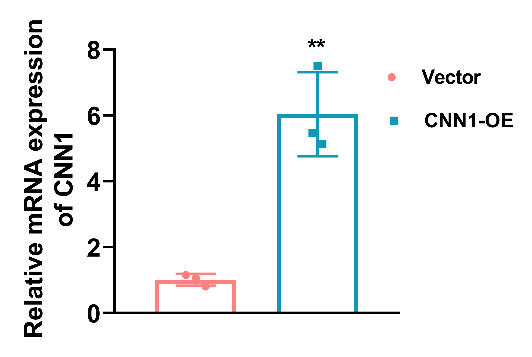


**Fig.S1** The overexpression efficiency of CNN1 overexpression plasmid (CNN1-OE) was confirmed via RT-PCR analysis using GAPDH as internal control. Data were presented as mean ± SD; **P < 0.01.


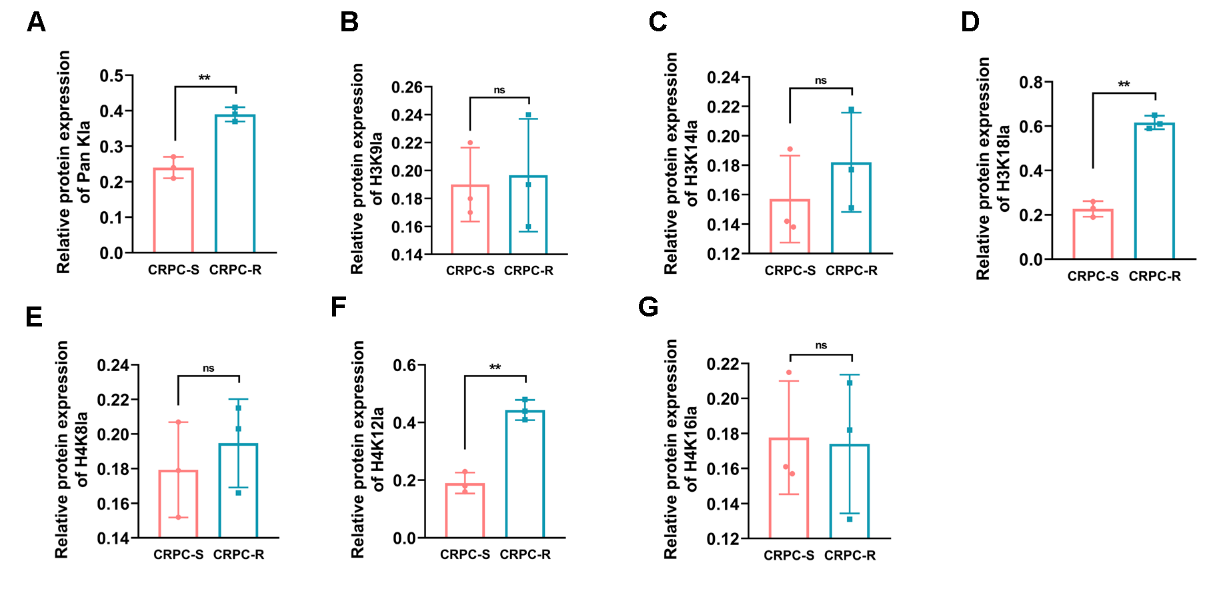


**Fig.S2** Quantification analysis of the protein levels of Pan Kla, H3K9la, H3K14la, H3K18la, H4K8la, H4K12la, H4K16la. Data were presented as mean ± SD; **P < 0.01, ns=non-significant.


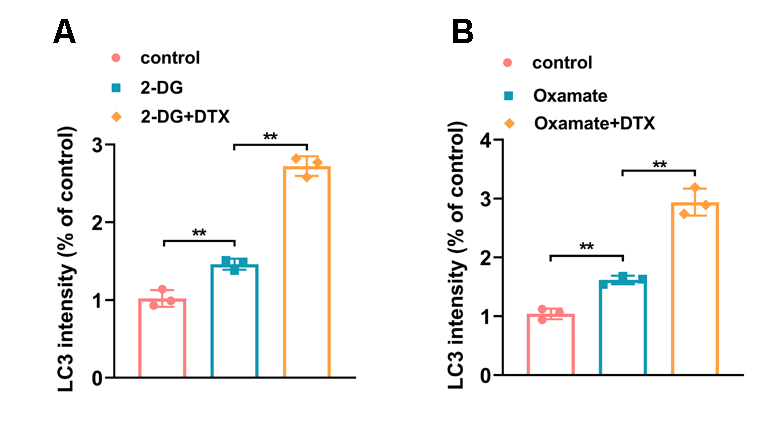


**Fig.S3** Quantification analysis of the expression of LC3. Data were presented as mean ± SD; **P < 0.01.


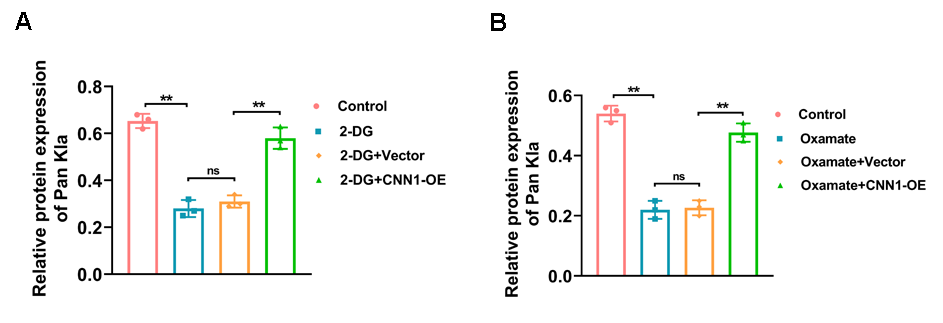


**Fig.S4** Quantification analysis of the protein levels of Pan Kla. Data were presented as mean ± SD; **P < 0.01, ns=non-significant.


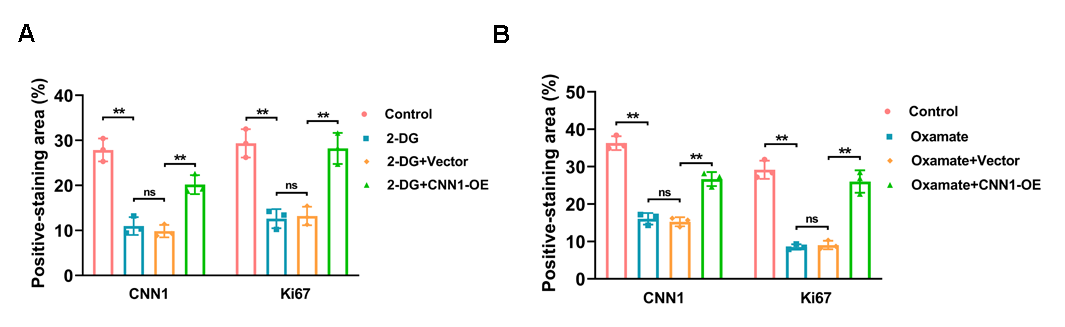


**Fig.S5** Quantification analysis of the expression of CNN1 and Ki67. Data were presented as mean ± SD; **P < 0.01.
