# Supplementary figures and images for: Histone lactylation modification promotes docetaxel resistance and tumor progression through CNN1-Mediated autophagy and cell cycle arrest in Castration-resistant prostate cancer
Source: Cell Death Discov. 2026 May 13;12:259. doi: 10.1038/s41420-026-03141-8 (PMC13221474; doi:10.1038/s41420-026-03141-8)

Fig.1E

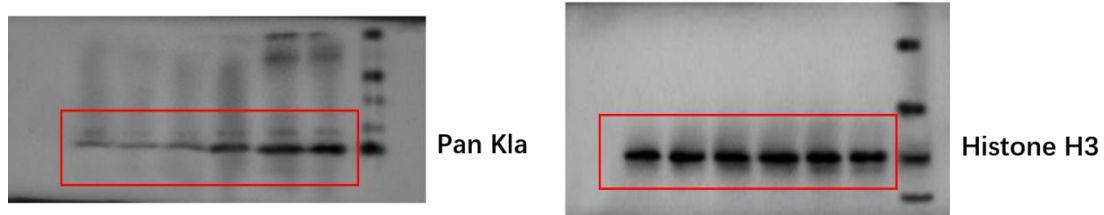

Fig.1H

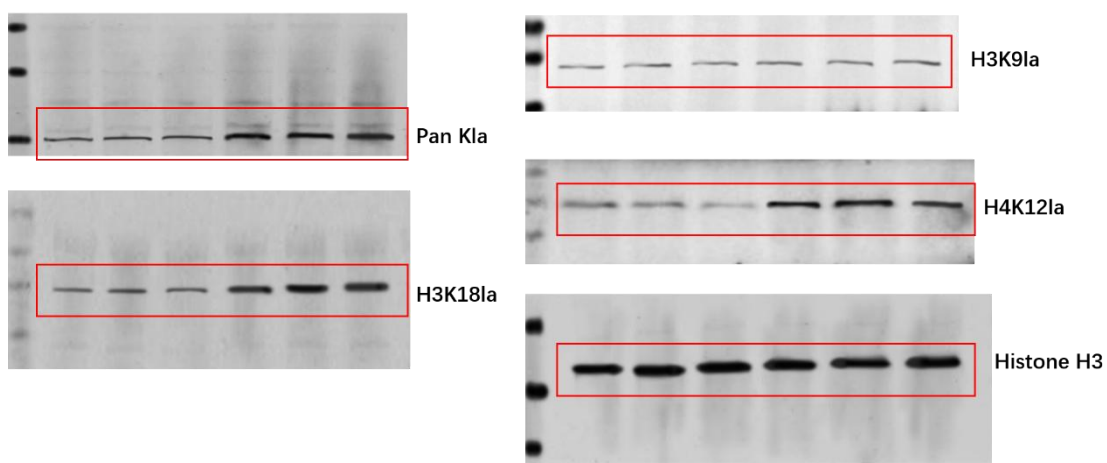

Fig.3G

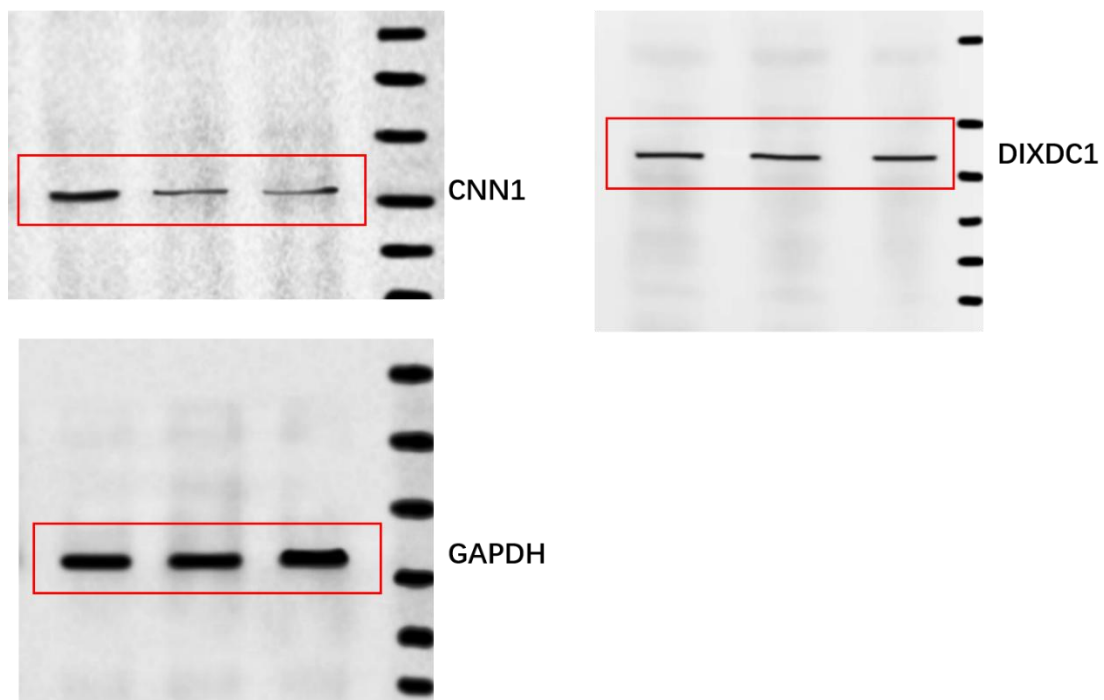

Fig.3O-P

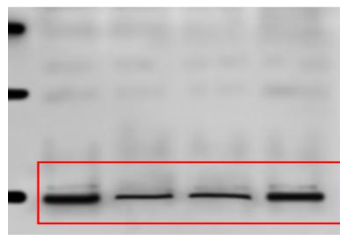

Pan KLa

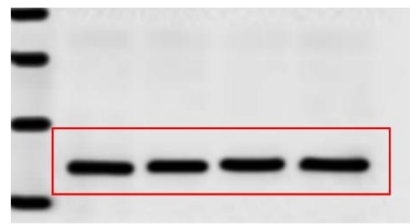

Histone H3

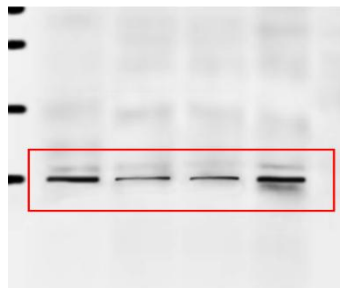

Pan KLa

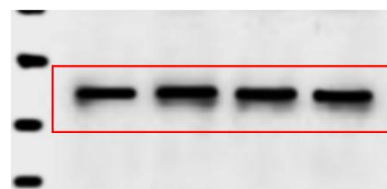

Histone H3

Supplement: Supplementary file 2 — original images of Western blots [file 41420_2026_3141_MOESM2_ESM.pdf]
